# Life Cycle Assessment of Flax Fiber Technical Embroidery-Reinforced Composite

**DOI:** 10.3390/polym17131888

**Published:** 2025-07-07

**Authors:** Andrzej Marcinkowski, Agata Poniecka, Marcin Barburski

**Affiliations:** 1 Institute of Marketing and Sustainable Development, Faculty of Organization and Management, Lodz University of Technology, ul. Żeromskiego 116, 90-924 Lodz, Poland; andrzej.marcinkowski@p.lodz.pl; 2 Institute of Architecture of Textiles, Faculty of Material Technologies and Textile Design, Lodz University of Technology, ul. Żeromskiego 116, 90-924 Lodz, Poland

**Keywords:** life cycle assessment (LCA), decision-making, sustainability, environmental management, composites, technical embroidery, tailored fibre placement (TFP), flax fibres

## Abstract

The aim of this study is to compare the environmental impact of composites reinforced with flax fiber technical embroidery and traditional woven fabric in order to provide conclusions supporting composite manufacturer management in making technology selection decisions. The research objectives are to identify the key stages in the life cycle of composites, from raw material acquisition to end-of-life; determine the environmental impact of each stage, with a particular focus on processes with the largest contribution to overall result; compare the environmental impact of embroidery-reinforced composites with traditional woven fabric-reinforced composites; propose strategies to minimize the negative environmental impact of composites, including modifying the component set and optimizing the production process. The method involves experimental research including the production of technical embroidery-based composites with varying stitch lengths and woven fabric-reinforced composites. The tensile strength of the composites was evaluated. Subsequently, life cycle assessment was conducted for each material according to the relevant ISO standards. The results presented in this paper provide a comprehensive assessment of the environmental performance of technical embroidery-reinforced composites and identify directions for future research in this field.

## 1. Introduction

The most common type of reinforcement in composites is woven fabric, especially those made from glass, carbon, or aramid fibers [1]. In traditional reinforcing woven fabrics, two perpendicular sets of yarns are interlaced, and the fabrics are layered in the composite. However, the low fatigue resistance and stiffness of 2D fabrics when subjected to shear stresses make their use challenging. Additionally, composites made from 2D structures are weaker and prone to delamination. As a result, the stability of the composite is limited to two, or at most three, axes, which is insufficient for many applications. Laminated composites made from woven fabric layers are susceptible to cracking and delamination, reducing their stiffness and strength. Delamination can occur during compression, tension, bending, and other loads during composite formation [2]. A solution to the problem of interlaminar cracking in composites and increasing their tensile strength is the use of technical embroidery for reinforcement. This technology provides bonding between layers using a zigzag stitch, thus preventing delamination.

Technical embroidery technology, known as tailored fiber placement (TFP), was developed at the Institute of Polymer Research Dresden in Germany in the 1990s [3]. It is an advanced method for producing composite materials, enabling the automated production of components with complex geometries that are optimally adapted to specific application requirements. This technology allows for the placement of the medium on the surface of a flat textile product in any direction, along the X and Y axes. By overlapping layers of embroidery, it is also possible to achieve a certain dimension in the Z-axis direction, although this is quite limited by the technical capabilities of the embroidery machine. TFP allows for the creation of any pre-designed pattern, which significantly reduces production waste. In the case of embroidery, the material cutting process is unnecessary, as a finished product is obtained. The only waste is the nonwoven fabric or other type of backing on which the embroidery is performed, and even this can be reduced by using appropriately sized hoops. TFP technology enables the production of materials with complex structures, where the fibers are distributed optimally, adapted to the loads they will be subjected to. By using TFP, it is possible to obtain components with very high strength and stiffness, while simultaneously reducing their weight and production costs [4].

A significant advantage of technical embroidery is the high-dimensional stability of the produced elements (thanks to the reinforcement of the structure with a securing thread in both the horizontal and vertical planes) and excellent repeatability of the production process. Due to the complete freedom of direction of the medium placement on the substrate offered by the computer-controlled embroidery machine, TFP can be fully customized. The automation of the embroidery process provides full control over pattern execution and raw material consumption [5].

Technical embroidery is used for the precise placement of fibers in a composite, allowing for the achievement of optimal mechanical properties. Desired composite properties are obtained by directing loads almost exclusively along the fiber orientation, minimizing shear stress on the matrix [6]. By using a fiber arrangement with a variable axis in the composite, better properties in terms of stiffness and strength can be obtained compared to traditional composites. The term VA refers to changing the fiber orientation at the layer level.

Desired composite properties are achieved by directing loads almost exclusively along the fiber orientation, thereby minimizing shear stress in the matrix. This method is particularly useful in the production of composites with complex shapes, where traditional fiber placement methods may be difficult or impossible [7,8].

Research conducted at the Institute of Polymer Research Dresden has demonstrated that thermoset composites containing holes created using TFP exhibit similar mechanical properties under cyclic tensile loading compared to composites reinforced with unidirectional fiber fabrics (UD fabric) [9]. Furthermore, customized fiber placement technology can contribute to the optimization of stress distribution along composite elements susceptible to damage [10]. However, all studies have been conducted using carbon, glass, or aramid fibers. The literature is lacking on composites produced using the TFP method with flax fibers. Therefore, the authors of this article decided to investigate this topic.

Technical embroidery is currently experiencing dynamic development, becoming a key element in creating innovative materials with a wide range of applications. Its growing popularity is due to several factors:

- Precision and personalization: Thanks to modern embroidery machines, it is possible to precisely arrange fibers at various angles, allowing for the creation of materials with precisely defined properties. This, in turn, enables the customization of the product to very specific customer needs.
- Lightweight and strength: Materials created on the basis of TFP combine low weight with high strength. This makes them ideal for applications in the aviation, automotive, and sports industries.
- Environmental aspects: The growing interest in environmentally friendly materials means that technical embroidery, especially when made from natural fibers, is gaining importance.

The authors have identified a gap in the existing literature regarding the use of TFP as a reinforcing element in composites, especially in the context of environmental impact. Although current papers on composites based on embroidery present applications of the TFP technology in light of potential environmental benefits [11,12,13], there are limited studies that determine the environmental impact of these materials. A global warming potential related to the specific stiffness for different natural and synthetic fibers in a composite component manufactured by TFP was investigated by the authors of [14]. The authors did not provide the environmental impact indicators for TFP-based materials in comparison to other technologies; however, the study pointed out certain premises suggesting the potential for reducing environmental impact through the use of embroidery: the possibility of precise fiber placement, which allows for optimal stiffness with less material use and waste minimization, and easy adaptation of the process to natural and synthetic fibers without major technological changes, facilitating the use of greener materials.

Given the literature gap identified, the main objective of this study is to determine and compare the environmental impact of a TFP-based composite (referred to as the TFP scenario) with a composite using woven fabric (referred to as the Fabric scenario). In line with this objective, the research question was formulated as follows: ‘Does the application of TFP in composite manufacturing provide a reduction in environmental impact compared to fabric-based composites, and if so, to what extent?’. In order to highlight the difference resulting from a fiber arrangement technology applied (technical embroidery vs. weaving), the same components were assumed to be used: flax fibers as a reinforcement, and an epoxy resin, applied using the infusion method, as a matrix. The analysis of environmental impact indicators alongside the strength parameters of various reinforcement technologies provides a practical reference for management staff in the composite industry, facilitating well-informed decision making regarding the selection of the most suitable technology.

## 2. Materials and Methods

### 2.1. Material Properties

**Figure 1:**
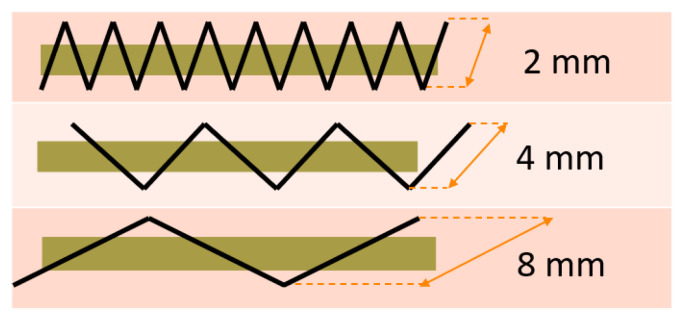
Visualization of the zig-zag stitch lengths used. Source: Own elaboration.

In this study, the focus was on tailored fiber placement made of Safilin’s flax roving with a linear mass of 400 tex. It was fastened with Gunold’s polyamide monofilament with linear mass of 11 tex. Three types of embroidery were made, differing in zig-zag stitch length: 2 mm, 4 mm, and 8 mm (see Figure 1). The width of stitch was 1.2 mm. The tensile strength of the flax roving was 7.37 cN/tex, and the tensile elongation was 1.69%. The embroidery was made on a base of cotton fabric with an area weight of 280 g/m^2^ and nonwoven fabric with an area weight of 35 g/m^2^. The flax-woven fabric was made from the same roving used for the embroidery. The surface mass of the fabric was 400 g/m^2^.

**Figure 2:**
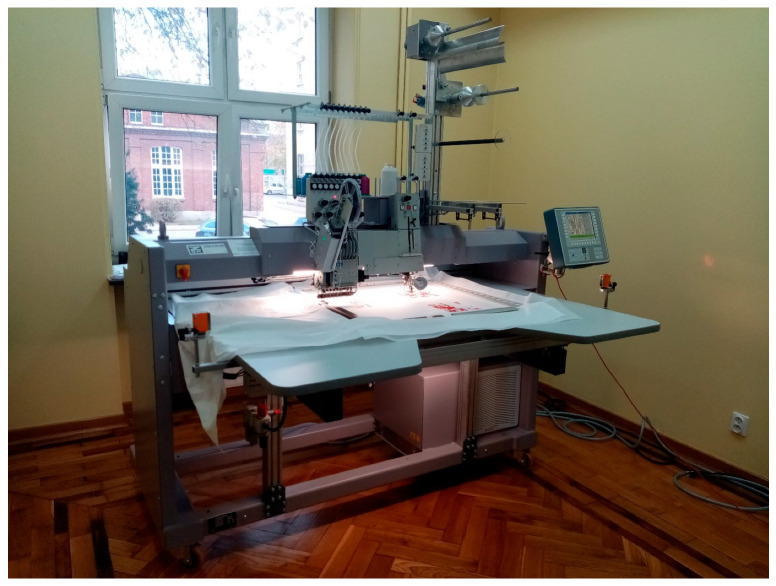
The embroidery machine ZSK model JCZA 0109-550. Source: Own photograph.

**Figure 3:**
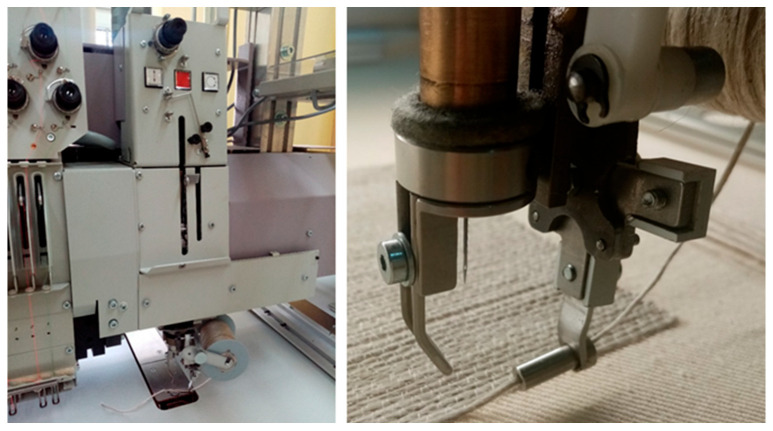
Head of the embroidery machine. Source: Own photograph.

**Figure 4:**
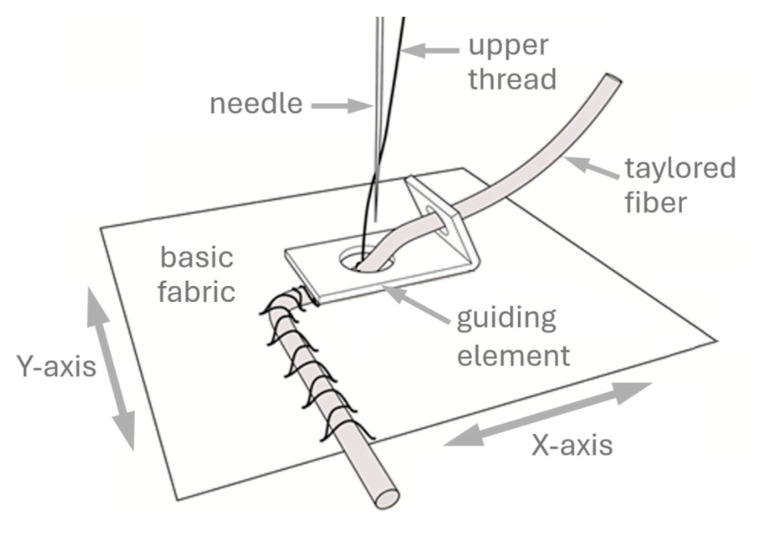
Scheme of laying the medium on the surface. Source: Own elaboration based on the study of [15].

The embroidery was performed using a ZSK computer-controlled TFP machine, model JCZA 0109-550 (ZSK Stickmaschinen GmbH, Krefeld, Germany), presented in Figure 2. This machine is equipped with a type W head (see Figure 3), which enables the arrangement of linear textile products (as well as cables, wires, optical fibers, and other continuous materials) according to a pre-designed pattern on a selected substrate. The scheme of laying the medium on the surface was presented in Figure 4. These products are attached to the substrate using a zigzag stitch with a securing thread. The maximum height of the layered assemblies can be 8 mm. The designs for the individual embroidery variants were created using the specialized software GiS BasePack version 10, dedicated to ZSK machines.

**Figure 5:**
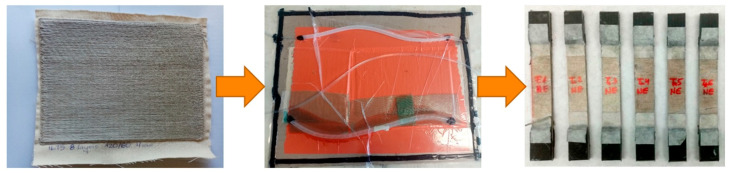
The stages of the composite production process: Dry sample-infusion composite. Source: Own elaboration.

The composites were made using infusion method with epoxy resin. The resin system consisted of SR GreenPoxy 33 epoxy resin and SD4772 hardener at a ratio of 100:32. The samples differed from each other in the arrangement of layers in the reinforcement. The variants considered are specified in Table 1. The characteristics of flax fiber materials used are provided by the manufacturer [16], while the technical data regarding the epoxy and hardener is available on the website [17]. A visualization of individual stages of the composites production process is depicted in Figure 5.

**Figure 6:**
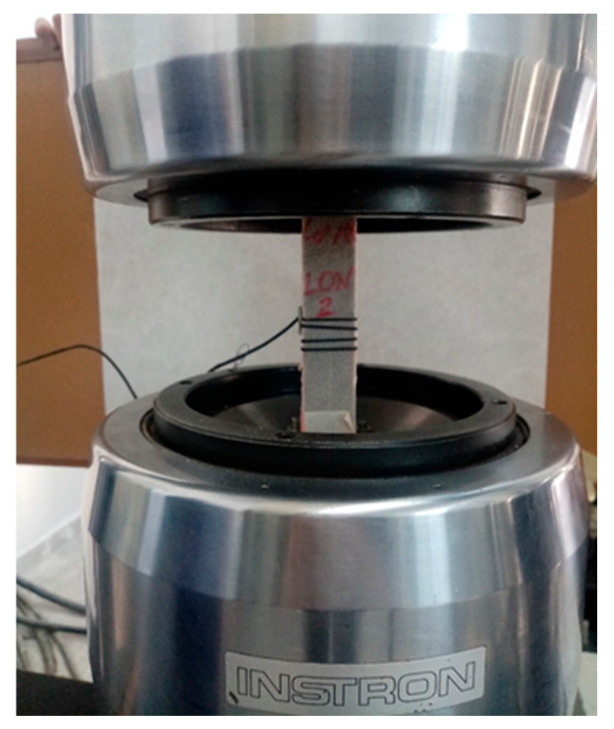
Tensile strength and tensile elongation test. Source: Own elaboration.

Test material was subjected to tensile strength tests on the INSTRON machine based on the ISO 527-4:2023 standard [18]. Test parameters were as follows: clamp distance: 100 mm; stretching speed: 1 mm/min; size of sample: 250 mm × 25 mm × 2 mm; and number of samples for each variant: 5. Full information about the test can be found in the article [19]. The photograph of the test of tensile strength and tensile elongation is presented in Figure 6.

### 2.2. The Environmental Impact Assessment

The environmental impact of the composites to be compared was determined with life-cycle assessment method in accordance with the standards [20,21]. The LCA framework consists of four phases: goal and scope definition, inventory analysis (LCI), impact assessment (LCIA), and interpretation. As the framework of LCA differs from the typical structure of scientific papers, the subsequent phases were included in the following sections of this study: Section 2, Section 3.

The boundaries of the systems were defined according to a cradle-to-grave approach. The product systems included natural resource extraction processes, transportation and processing of raw materials, electricity generation, and waste disposal. The functional unit was defined as 1 m^2^ of the composite materials, which served as a bicycle saddle with dimensions 182 × 277 mm and an area of 280 cm^2^. The main material of the reinforcement was flax technical embroidery and flax-woven fabric. The matrix was the epoxy resin applied with infusion method. The source of the detailed material and energy balance data of the production processes considered in the analysis was the ecoinvent 3.8 database [22]. However, the technical specifications for TFP process, including components used and electricity consumption, were provided by the authors, based on their own laboratory measurements.

The development of product system models required a number of assumptions presented below.

- The matrix manufacturing, including materials, electricity consumption, and waste disposal, were excluded from the product systems boundaries as elements that were identical for both the compared scenarios. This was justified by provisions of System boundary section of ISO 14044 [21], which says that exclusion of given processes is permitted if it does not significantly change the general conclusion of the analysis.
- The reinforcement based on TFP consisted of the following: nonwoven polyester with an area weight of 35 g/m^2^, cotton fabric with an area weight of 280 g/m^2^, flax roving with a linear weight of 400 tex, and polyamide monofilament with a linear weight of 11 tex.
- The reinforcement based on the fabric was made from the same flax roving as used for embroidery manufacturing (400 tex), and the resulting area weight of the fabric was 400 g/m^2^.
- Obtaining the irregular shape of a bicycle saddle requires trimming from a rectangular piece of the fabric, which results in waste with a proportion of 0.496 m^2^ per 1 m^2^ of raw material used. In the case of TFP, the flax fibers were arranged directly into the desired shape. The waste (with the same proportion) was therefore only the nonwoven fabric and the cotton fabric on which the embroidery was made.
- The production of 1 m^2^ of TFP reinforcement requires the consumption of 2.052 kWh of low-voltage electricity according to the mix specific for European countries.
- As the means of transport of cotton fabric, a railroad freight was assumed with transportation distance of 12,000 km, as the estimated distance between East Asian woven cotton producer and the reinforcement manufacturing site.
- As the means of transport of flax fiber, nonwoven polyester, and polyamide monofilament, a road freight was assumed, and specifically, lorry of the size class 16–32 metric tons gross vehicle weight meeting EURO4 emission standard was operated under European conditions. The transportation distance was assumed to be 1500 km as the estimated distance between European component producers and TFP manufacturing site.
- The method of waste material disposal at the end-of-life phase was waste incineration, as one of the main current waste management strategies for most fiber-reinforced composite materials [23].
- The lifespan of compared composites was assumed to be the same.
- The allocation was based on physical (mass) principle.

Table 2 demonstrates the input dataset calculated on the basis of the assumptions made. The presented data represent amounts corresponding to the functional unit. From a production management perspective, the data, alongside a set of underlying assumptions, allow for the development of a plant-specific methodological approach to assess the technologies being compared in terms of raw material and energy consumption, supporting both operational and strategic planning.

The life cycle impact assessment was carried out applying ReCiPe Midpoint (H) method resulting in characterization indicators. Furthermore, the endpoint indicators were also determined using ReCiPe Endpoint (H) method [24]. The indicators reflect effects on the environment in three damage categories: human health, ecosystems, and resources. Each of the categories has its specific unit. The unit of human health category is disability adjusted life-years (DALY), representing the years of life that are lost or that a person is disabled due to environmental issues. The damage to ecosystems is expressed by local species loss integrated over time (species year). The unit for the category of resource scarcity is the dollar (USD), reflecting the surplus costs involved for future extraction of mineral and fossil resources [25]. In the following sections of this study, the endpoint indicators, initially determined in their specific units, were expressed in percent, showing the relative proportions between the results. Depicting percentage indicators allowed us to see the results in the same order of magnitude. Otherwise, it would be impossible to observe the comparison between the environmental effects exerted within individual categories, as the differences between the respective indicators reached 6 orders of magnitude.

## 3. Results and Discussion

### 3.1. Material Properties

**Figure 7:**
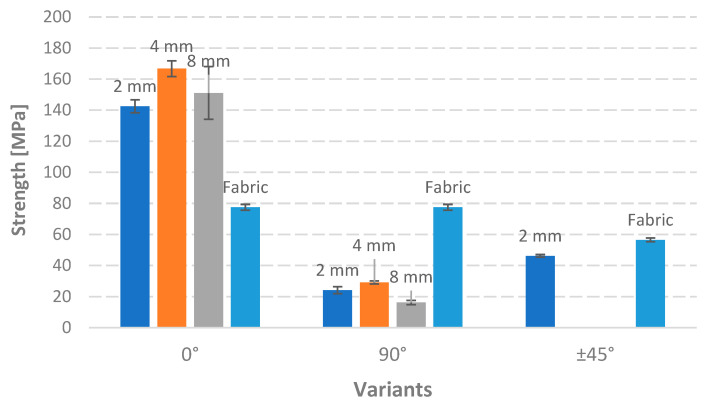
Strength of the composite variants specified in Table 1. Source: Own research.

The maximum strength of produced composites is presented in Figure 7. The woven fabric-reinforced composite exhibited significantly higher strength than all other 90-degree variants. This is attributed to the fabric’s woven structure, where half of the threads are aligned with the loading direction, contributing significantly to load bearing. Embroidery offers precise control over fiber placement, allowing for optimized strength by aligning fibers closer to the 0-degree direction of the expected load. The orientation of the reinforcement significantly influences the tensile strength of the composites. TFP can effectively enhance the strength of composites, even when the reinforcement is oriented perpendicular to the loading direction. The structure and arrangement of the reinforcement material play a crucial role in determining the overall mechanical properties of the composite.

### 3.2. The Environmental Impact Assessment

**Figure 8:**
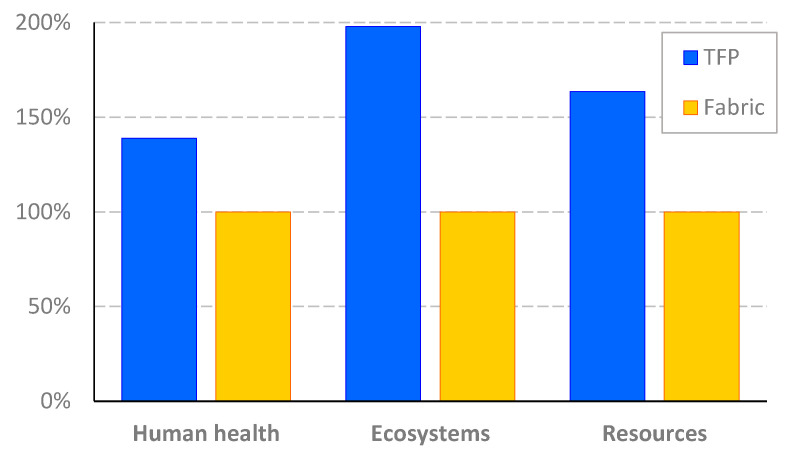
Relative endpoint indicators for TFP and fabric-based composites. Source: Own research.

Table 3 presents midpoint characterization indicators for the compared scenarios. For all the impact categories considered, the TFP-based composite demonstrates higher environmental impact compared to the fabric-based one. This observation is confirmed by Figure 8, which depicts relative damage indicators obtained for both scenarios (100% refers to the impact of the Fabric scenario). For the TFP scenario, the results were higher by 39–98% than for the Fiber scenario, depending on the damage category considered. The greatest difference was identified in the damage to ecosystems.

**Figure 9:**
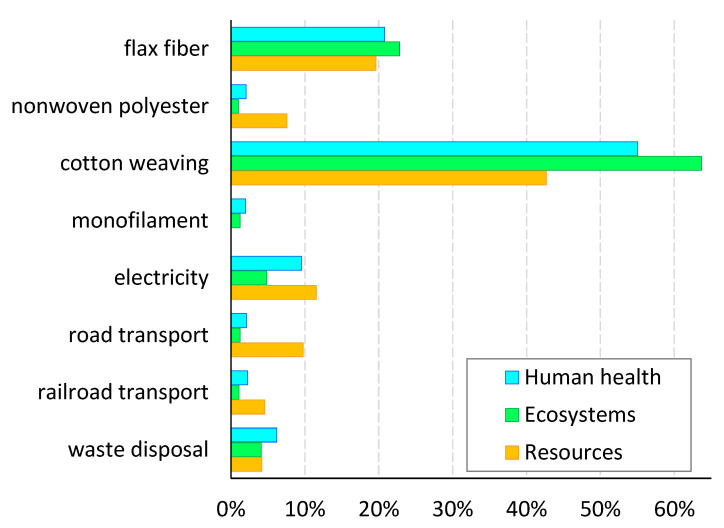
Contribution to total environmental damage by individual processes of TFP-based product. Source: Own research.

**Figure 10:**
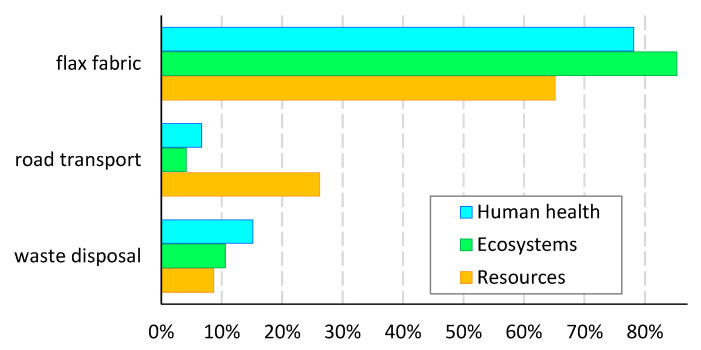
Contribution to total environmental damage by individual processes of fabric-based product. Source: Own research.

In order to analyze the main reasons for environmental impact attributable to the TFP-based product, a comparison of relative endpoint indicators for involved processes was determined and presented in Figure 9, where 100% refers to the total impact of the TFP scenario. The greatest contribution to the environmental damage resulted from the cotton weaving production process, representing 43–64% of the total environmental burden. Comparatively, the processes of monofilament and nonwoven polyester production, as well as transportation and waste disposal, indicated a contribution of only up to 6–10% each, depending on the category considered. An analogous breakdown prepared for the flax fabric-based saddle was presented in Figure 10, where 100% refers to the total impact of the Fabric scenario.

**Figure 11:**
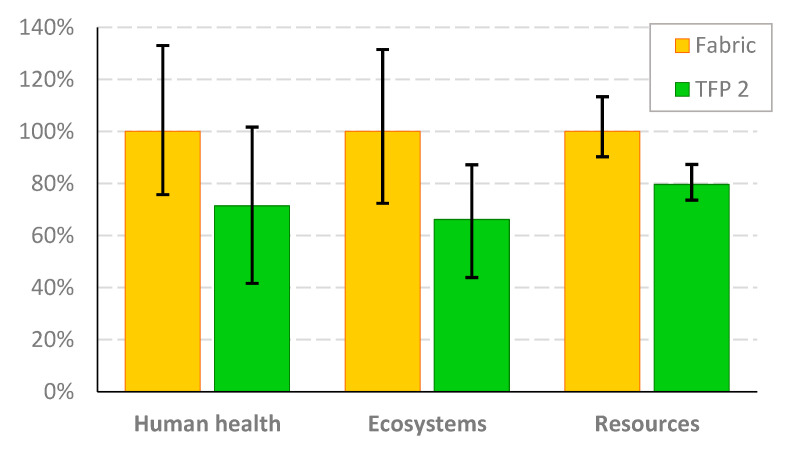
Relative endpoint indicators for TFP2 and fabric-based composites. Source: Own research.

Identifying the element of the production system most responsible for the overall impact provides a strong foundation for product improvement in terms of strategic decision making in supply chain management, optimizing resource allocation, and exploring alternative materials with lower environmental impact. Analysis of the role of cotton fabric revealed that its use is not obligatory for the production of TFP-based reinforcement. Its application improves the stability of the embroidery, but it can only be made on a nonwoven polyester backing. For this reason, the reanalysis of the TFP-based saddle was carried out, eliminating cotton weaving from the inventory. In order to differentiate from the analysis presented above, the revised product assumptions are referred to as the TFP2 scenario. Figure 11 depicts the comparison of relative endpoint indicators for TFP2 and Fabric scenarios. The 100% level on the vertical axis reflects the relative impact of fabric-based products. In comparison, the TFP2 indicators significantly decreased by the proportion attributable to the cotton fabric impact. A remarkable fact is that the proposed change in the technical embroidery technology provided the environmental effect that turned out to be lower than the impact of the fabric-based scenario. The differences in the indicators depended on the damage category considered and amounted to 29% for human health, 34% for ecosystems, and 20% for resources.

The demonstrated environmental superiority of the TFP2 scenario over the Fabric scenario, in the absence of mechanical validation of the TFP2 composite, must therefore be regarded as a conditional advantage rather than an inherent one. However, the differences in tensile strength measured along different directions (see the 0°, 45°, and 90° variants in Figure 7) suggest a relatively minor contribution of the cotton fabric to the overall strength of the composite. In the case of fabric-reinforced composites, the variation in tensile strength across these orientations reaches approximately 27%, whereas, for the TFP-based products, the difference is as high as 83%. Although direct experimental confirmation of this hypothesis has not been carried out, the reported test results strongly support its validity. Removing the cotton weaving would certainly weaken the structure of the composite; however, even under a pessimistic assumption that, in the 90° orientation, the tensile strength derives solely from the cotton fabric and not at all from the TFP structure, it can be inferred that, in the 0° direction, the fabric does not contribute substantially to the overall strength of the material. The results obtained suggest that the environmental impact differences between the Fabric and TFP2 scenarios are greater than the corresponding differences in tensile strength that can be expected.

Although the mechanical performance of the TFP2 composite remains uncertain and requires further experimental validation, its potential environmental advantages cannot be viewed in isolation. While the LCA results suggest clear benefits over the Fabric scenario, these gains must be weighed against practical considerations surrounding manufacturability. Implementing the TFP2 structure at an industrial level could introduce new challenges, such as stricter tolerances during fiber placement, longer production cycles, and the need for specialized machinery—all of which could increase production costs. Furthermore, the scalability of the process must be proven under real-world conditions to ensure that any environmental improvements can be achieved without compromising structural integrity or economic viability. Future work should therefore not only focus on confirming the material’s strength but also on evaluating the feasibility of producing TFP2 at scale to support informed decision making about its adoption in commercial practice.

Uncertainty analysis of the input parameters in the LCA model was carried out using a Monte Carlo simulation, with 5000 iterations. Mean indicators, 5% and 95% percentiles, as well as standard deviations (SD), depending on the endpoint category, are presented in Table 4. The ranges specific to the 5% and 95% percentiles are also marked in Figure 5 with vertical lines.

In order to identify critical input parameters and test initial assumptions, the sensitivity analysis was carried out. The crucial input in the case of TFP-based reinforcement turned out to be the application of cotton fabric. As described above, eliminating this auxiliary component resulted in a significant reduction in the environmental damage and changing the general conclusion that could be drawn from the comparison of the embroidery-based composite and the fabric-based one. Another element significantly affecting the total environmental impact is flax fiber, with the contribution ranging 20–23% in the TFP scenario and 38–67% in the TFP2 scenario. However, this is the essential component of the reinforcement underlying the strength of the whole structure. An analogous fundamental element in the Fabric scenario is flax fabric contributing 65–85% to the overall impact. Reducing the amount of flax material would adversely affect the product performance; therefore, any change is not recommended in this case.

According to initial assumptions, the low-voltage electricity mix specific to European countries was adopted in TFP and TFP2, as the technical embroidery was performed in a Central European country. Within the sensitivity analysis, the effect of different electricity mix specific for individual countries could be examined; however, since this process contributes only 5–12% of the total impact of TFP-based scenarios, the effect would be limited. Moreover, in the case of the Fabric scenario, the electricity consumption was inbuilt in the flax fabric manufacturing process of the ecoinvent database in line with the site-specific mix. For this reason, this would be difficult to change this assumption in the Fabric scenario in line with TFP-based cases. The potentially obtained sensitivity results could be misleading.

Transportation processes in the TFP scenario contributed only 1% (Ecosystems) up to 10% (Resources) to the total endpoint indicator and only below 5% (Resources) in the case of railway transport of cotton fabric. For the transport of goods from a East Asian country to Central Europe, an oceanic freight could be considered instead of railroad transport. The replacement of train transport with oceanic transport would result in a longer transport distance but a significantly lower environmental impact per ton-kilometer (t·km). However, it seems there is no reason to analyze such an assumption since cotton fabric and consequently its transport were eliminated from the TFP2 inventory.

The remaining components of the analyzed products were assumed to be transported by road vehicles with a gross vehicle weight of 16–32 t meeting the EURO4 emission standard. The greatest contribution of 10% to total TFP environmental damage occurred for the Resources category, whereas, for human health and ecosystems, contributions measured 2% and 1%, respectively. This consideration also concerned the Fabric scenario, where the same transportation means were assumed to transport flax fabric. In this case, the road transport contributed as much as 26% to the total damage in resources. The significant impact on resources resulted from fuel consumption by lorries and could be reduced by replacing the fleet with vehicles meeting higher emission standards (EURO5). This change in the assumption would be expected to improve the environmental performance of transport and consequently the entire impact of TFP and Fabric scenarios. In order to examine the influence of the improved emission standard on the results, an additional analysis was carried out. The determined LCA indicators assuming EURO5 showed a 4–5% reduction in road transport impact in the human health and ecosystem categories. However, in the case of the Resource category, where the reduction was expected, the results indicated an increase in the environmental damage. This paradox can be explained by concepts of advanced strategies to control nitrogen oxide emissions by lowering combustion temperatures, which reduces NO_x_ formation but inevitably increases fuel consumption [26].

The distance between the European component manufacturers (primarily located in France or Benelux) and the technical embroidery production site (situated in Central Europe) was assumed to be 1500 km, by estimation. Modifying this assumption results in a proportional change in the environmental impact. If the distance were 750 km, the impact would be reduced by 50%. Given the contribution of this process to total impact (1–10% in TFP, 5–19% in TFP2 and 7–26% in Fabric), the potential of changing the overall results by assuming a shorter distance does not exceed a few percent, with the exception of the Resources category in TFP2 and Fabric scenarios, where the change in whole impact might exceed 10%.

Replacing road transport with train freight was analyzed as an alternative scenario too. The endpoint indicators determined demonstrate a significant reduction in the environmental damage, with a range of 60–84% depending on the individual category with maximum reduction attributable to Resources. Given the transportation process contribution to the entire impact, the lower emission intensity of rail transport, and its high energy efficiency, this change could lead to a moderate reduction in impact. However, logistical constraints and infrastructure availability may limit the feasibility of such a transition in real-world applications.

## 4. Conclusions

Given the main objective of this study, it might be concluded that the application of embroidery in composite manufacturing has significant potential for reducing the damage in all endpoint categories. These opportunities resulted in lowering the environmental impact of TFP-based composite, supporting efficient resource management and decision making in material selection. This study confirms that TFP-based composites exhibit superior tensile strength compared to fabric-based composites. However, the environmental burden associated with customized fiber placement, primarily due to cotton fabric inclusion, requires a careful trade-off analysis. The LCA results highlight that cotton weaving significantly contributes to the environmental impact of TFP-based composites. Eliminating cotton fabric from the TFP reinforcement structure led to a substantial reduction in the environmental burden, shifting the results in favor of TFP2 over traditional woven fabric-based composites. This finding suggests that modifications in TFP composite design can enhance its environmental performance without compromising mechanical properties. This superiority must be regarded as a conditional advantage until TFP2 is fully validated mechanically, although the differences in tensile strength across orientations imply that the removal of cotton fabric yields greater environmental benefits than any potential losses in composite strength. To summarize, this study provides valuable insights into the environmental impact of TFP- and woven fabric-based composites, offering a decision-making advantage to the management of composites manufacturers; however, certain limitations must be addressed in future research. The assumption that the removal of cotton fabric does not significantly affect mechanical performance must be validated through experimental tensile strength measurements.

## Figures and Tables

**Table 1:** Specification of the composite variants considered.

Variant	Name	Graphic Orientation of Roving in Each Layer	Area Mass of Dry Sample [g/m^2^]	Area Mass of Composite [g/m^2^]	Volume Fraction [%]
Embroidery 0° 2 mm	0° 2 mm	││││	1424	3439	41
Embroidery 0° 4 mm	0° 4 mm	││││	1273	3318	38
Embroidery 0° 8 mm	0° 8 mm	││││	1227	3561	34
Embroidery ±45° 2 mm	±45° 2 mm	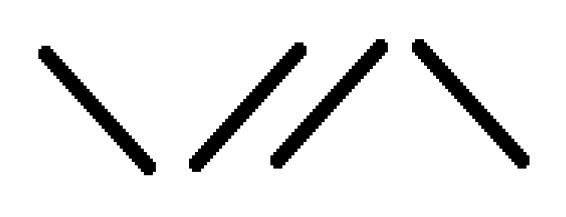	1387	3425	40
Embroidery 90° 2 mm	90° 2 mm	─ ─ ─ ─	1515	5015	30
Embroidery 90° 4 mm	90° 4 mm	─ ─ ─ ─	1303	3455	38
Embroidery 90° 8 mm	90° 8 mm	─ ─ ─ ─	1227	3561	34
Woven fabric 0°/90°	Fabric 0°/90°	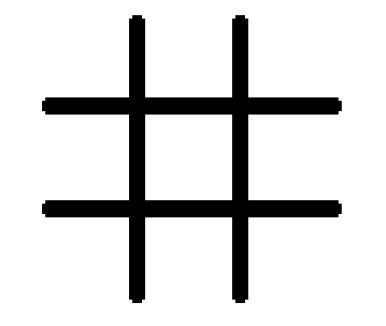	1802	4309	42
Woven fabric ±45°	Fabric ±45°	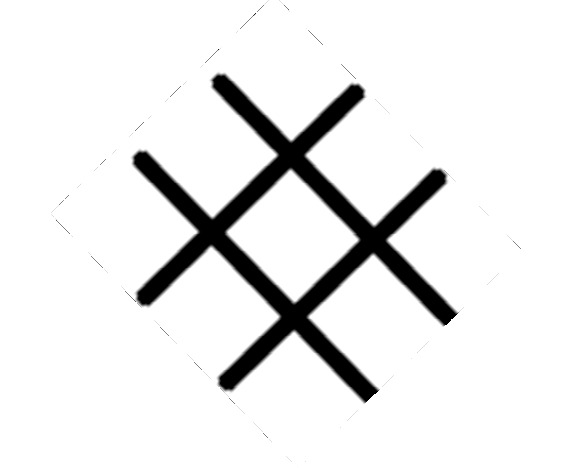	1867	4254	44

Source: Own elaboration.

**Table 2:** LCI datasets for the compared scenarios.

Component	TFP	Fabric
Flax fibers	886 g/m^2^	
Cotton fabric	280 g/m^2^	
Nonwoven polyester	35 g/m^2^	
Polyamide monofilament	26.7 g/m^2^	
Flax fabric		1600 g/m^2^
Low-voltage electricity consumption	2.052 kWh/m^2^	
Railroad transport	3.36 t·km	
Transport by lorry 16–32 t	1.422 t·km	2.40 t·km
Waste textile material	1227.7 g/m^2^	1600 g/m^2^

Own elaboration based on ecoinvent 3.8 processes are used for European conditions where available.

**Table 3:** Midpoint characterization indicators for TFP and fabric-based composites.

Impact Category	Unit	TFP	Fabric
Fine particulate matter formation	kg PM2.5 eq	0.0147	0.0082
Fossil resource scarcity	kg oil eq	1.369	0.577
Freshwater ecotoxicity	kg 1,4-DCB	0.394	0.067
Freshwater eutrophication	kg P eq	3.39 × 10^−3^	6.79 × 10^−4^
Global warming	kg CO_2_ eq	7.475	3.937
Human carcinogenic toxicity	kg 1,4-DCB	0.307	0.056
Human non-carcinogenic toxicity	kg 1,4-DCB	4.172	−3.837
Ionizing radiation	kBq Co-60 eq	0.682	0.0036
Land use	m^2^a crop eq	2.697	0.573
Marine ecotoxicity	kg 1,4-DCB	0.423	0.070
Marine eutrophication	kg N eq	0.0198	0.0082
Mineral resource scarcity	kg Cu eq	0.0180	0.0061
Ozone formation, human health	kg NO_x_ eq	0.0258	0.0170
Ozone formation, terrestrial ecosystems	kg NO_x_ eq	0.0262	0.0172
Stratospheric ozone depletion	kg CFC11 eq	4.84 × 10^−5^	4.11 × 10^−5^
Terrestrial acidification	kg SO_2_ eq	0.0536	0.0363
Terrestrial ecotoxicity	kg 1,4-DCB	20.170	2.060
Water consumption	m^3^	2.017	0.799

Source: Own research.

**Table 4:** Uncertainty analysis parameters for TFP2 and Fabric scenarios.

Endpoint	TFP2 Scenario			
	Mean	SD	5%	95%
Human health	7.1 × 10^−6^	1.23 × 10^−6^	4.14 × 10^−6^	1.0 × 10^−5^
Ecosystems	2.4 × 10^−8^	3.26 × 10^−9^	1.62 × 10^−8^	3.2 × 10^−8^
Resources	1.4 × 10^−1^	8.01 × 10^−3^	1.28 × 10^−1^	1.5 × 10^−1^
Endpoint	Fabric scenario			
	Mean	SD	5%	95%
Human health	9.9 × 10^−6^	1.21 × 10^−6^	7.54 × 10^−6^	1.3 × 10^−5^
Ecosystems	3.7 × 10^−8^	4.55 × 10^−9^	2.67 × 10^−8^	4.9 × 10^−8^
Resources	1.7 × 10^−1^	1.29 × 10^−2^	1.58 × 10^−1^	2.0 × 10^−1^

Source: Own research.

## Data Availability

All data used during this study appear in the submitted article.
